# “There is no peace when you are excluded”: exploring peace and peacebuilding with children and youth affected by armed violence

**DOI:** 10.3389/fpsyg.2025.1689758

**Published:** 2025-11-18

**Authors:** Catherine Baillie Abidi

**Affiliations:** Department of Child and Youth Study, Mount Saint Vincent University, Halifax, NS, Canada

**Keywords:** children and youth, peace and security, armed violence, peace perspectives, peacebuilding, children’s rights, participation

## Abstract

The global community is becoming increasingly fragile, plagued with intensifying armed violence, fracturing democratic governance processes, diminishing commitments and actions to protect basic human rights, and raging climate crises. Amid these complexities, children and youth are disproportionately impacted, and all signs point to increased precarity of their rights. Young people affected by armed violence are particularly suffering, thus understanding their rights and needs during and post conflict is essential to building effective peace and security. This research features the peace perspectives of 50 children and youth, all impacted by armed violence, and demonstrates the power of listening to young people’s visions for peace. The key findings illustrate the importance of interpersonal peace, learning peacebuilding skills, and the essential role that children and youth play in building peace in their homes, schools, and communities. Recommendations for meaningful child engagement in the development of a Children, Peace, and Security Agenda are featured.

*“Peacebuilding is a long-term investment, and we’re just at the beginning.”*

- Ilwad Elman, 2019 Nobel Peace Prize Nominee

## Introduction

The global community is becoming increasingly fragile, plagued with intensifying armed violence, fracturing democratic governance processes, diminishing commitments and actions to protect basic human rights, and a raging climate crisis. Amid these complexities, children and youth are disproportionately impacted, and all signs point to increased precarity of their rights. Children and youth are developing at a time of heightened insecurity and depleting hope, a time when learning peacebuilding skills and principles is constrained by pervasive inequitable systems and structures, and the consequences of these layered impacts of violence are oppressing generations. Young people affected by armed violence are particularly suffering, physically, emotionally, and mentally. They are forcibly displaced, persecuted, and detained. They are separated from and lose loved ones. Their schools are targeted, closed, and in some cases, permanently destroyed. They are increasingly impacted by poverty and face bleak employment prospects for healthy and sustainable futures. Moreover, they face a systematic unwillingness among adults to listen to, value, and collaborate across a range of social issues. Yet, young people are also fierce peace activists—in their homes, their schools, and their communities—leading important peace processes and demanding change. They are learning their rights, naming their needs, and identifying strategies for peace. This research features the peace perspectives of 50 children and youth, all impacted by armed violence, and demonstrates the importance of listening to young people’s visions for peace.

### Children and the current state of the world

The number of children living in conflict-affected regions continues to grow at a distressing rate, with over 473 million living in conflict zones (Østby and Rustad, 2024) and nearly 50 million displaced by violence, internally and internationally (United Nations Children’s Fund, 2025). The annual United Nations Secretary-General (2025) report on children and armed conflict illustrates an alarming trend of increasing grave violations and a fracturing of respect for the protection mechanisms designed to keep children safe and well. Grave violations against children include maiming and killing, sexual violence, recruitment, abduction, attacks on schools or hospitals, and the denial of humanitarian access, and these violations are monitored annually through the UN Monitoring and Reporting Mechanism (MRM) as directed through Security Council Resolution 1612 (United Nations Security Council, 2005). The 2025 report revealed a disturbing 25% increase in validated grave violations committed against children, totaling 41,370, with some children experiencing multiple violations (United Nations Secretary-General, 2025). The regions with the largest number of validated violations included Israel and the Occupied Palestinian Territory, the Democratic Republic of Congo (DRC), Somalia, Nigeria, and Haiti (ibid). The levels of violence documented against children are unprecedented, are compounded by health and environmental crises, and are influenced by intensifying global inequities, which are being exacerbated by a rise in right-wing essentialism, seen, for example, in recent decisions to drastically cut global humanitarian assistance. These intersecting sites and sources of harm are shattering the protection of children’s rights and deepening their needs.

Children’s experiences living in contexts of armed violence have always been fraught with danger and harm, a reality Graça Machel alerted the international community to in her seminal report in 1996, which acknowledged the disproportional impact of armed violence on children (Machel, 1996). Alarmingly, state and non-state actors are increasingly targeting children, and these trends are being documented across conflict regions. The United Nations Secretary-General (2025) annual report on children and armed conflict indicated “warfare strategies included attacks on children, the deployment of increasingly destructive weapons, the use of explosive weapons in populated areas and the systematic exploitation of children for combat” (p. 2). The Independent International Commission of Inquiry, tasked by the Human Rights Commission to investigate alleged violations of international humanitarian and human rights law in the Occupied Palestinian Territory and Israel, argued that, in addition to the deeply disturbing killing of over 15,600 Palestinian children, nearly 660,000 children are not able to access formal education (Office of the High Commissioner for Human Rights, 2025). The report further argues that “the destruction of the education system in Gaza is expected to harm Palestinians for generations to come, with consequent challenges in economic development, work and social abilities” (Office of the High Commissioner for Human Rights, 2025, p. 6). In the DRC, sexual violence against girls is particularly troubling (United Nations Secretary-General, 2025), bringing attention to the gendered impacts of armed conflict on children and the ways in which social identities and power structures intersect to particularly disadvantage marginalized children. In Sudan, the displacement of thousands of children due to intercommunal violence illustrates how generational cycles of violence, sustained through inequitable structures and systems, intensify children’s needs and lead to barriers to building peace. Furthermore, the millions of children forcibly displaced by conflict around the world also face many barriers, from family separation to lack of access to basic health and social services, to heightened vulnerability to abuse and exploitation (Hadfield et al., 2017; United Nations High Commissioner for Refugees, 2016). Children remaining in conflict-affected regions, as well as those displaced by conflict, have complex and diverse experiences. Yet, their voices are largely missing from the development, implementation, and evaluation of peace and security policies and practices.

### Conceptualizing children and protection

Though the definition of a child can vary regionally and culturally, for the purposes of this study, a child is defined as “every human being below the age of 18” (UNCRC, 1989). Utilizing this definition is in line with special protections afforded to children in international humanitarian and human rights law, such as preventing the recruitment and use of children in armed conflict and ensuring access to humanitarian aid. These special protections are embedded in instruments such as the Geneva Conventions (International Committee of the Red Cross, 1949), the Convention on the Rights of the Child (UNCRC, 1989), and the African Charter on the Rights and Welfare of the Child (Organization of African Unity, 1990). In addition, there are many political frameworks that seek to enhance protections for children affected by armed conflict in recognition of their unique needs, including the Paris Commitments to Protect Children Unlawfully Recruited or Used by Armed Forces or Armed Groups (“The Paris Principles”) (United Nations Children’s Fund, 2007), the Safe Schools Declaration (The Global Coalition to Protect Education from Attack, 2015), and the Vancouver Principles on Peacekeeping and the Prevention of the Use of Child Soldiers (Government of Canada, 2017). Together, these protection mechanisms bring attention to the unique vulnerabilities children face in armed conflict and set guidelines for reducing harm.

Protecting children from direct forms of violence has been the main and important focus of international protection mechanisms. Given the gap in recognition of children’s centrality to peace and security, the aforementioned frameworks and political commitments are essential. However, two key challenges require further consideration. First, except for the Geneva Conventions, these frameworks sit on the periphery of the peace and security architecture, demonstrating the minimalization of children’s significance to broader peace and security. This reduced visibility is evident in the lack of a Children, Peace, and Security agenda (Johnson et al., 2023) and, for example, consideration for children in conflict early warning (Baillie Abidi and Cleave, 2023). Failing to monitor early warning indicators related to children in fragile and conflict contexts means, in some cases, that nearly 50% of the population is not factored into conflict prevention, assessment, and response policy and practice. Second, these mechanisms have been designed by adults *for* children, often reinforcing the narrative that children affected by armed violence are passive and helpless victims (Denov, 2012; Drumbl, 2012; Johnson et al., 2022; Tabak, 2020), and that children’s perspectives and lived experiences are inferior to those of adults. These conceptualizations have complicated and, in most cases, overlooked children’s role in peace and security and children’s agency within armed violence. Children’s lack of perceived agency has contributed to their peripheral recognition in peace and security.

## Theoretical and methodological frameworks

Peace and violence are deeply interconnected concepts that exist along a complex continuum. According to Galtung (1969, 1996), violence can be understood as three interrelated forms: direct (physical and visible violence, such as hitting or hate speech), structural (exploitive and inequitable systems, such as racist, anti-sematic, or Islamophobic laws or policies), and cultural (normalizations of violence, such as values that dehumanize certain groups of people in our communities). The violence triangle, as the three elements are dubbed, aims to critically expose how social norms enable violence to manifest and sustain (Galtung, 1996). Breaking generational cycles of violence requires a deep dive to explore how violence was and continues to be socially constructed. The social construction of violence has long been asserted by scientists (United Nations Educational, Scientific and Cultural Organization, 1989; World Health Organization, 2002) who emphasize that humans appear to have an inherent propensity for cooperation.

While the international community has systems to monitor direct violence perpetrated against children, such as the UN MRM, and these systems are important to track the impacts of violence, deeper analyses that aim to critique and challenge the normative frameworks enabling violence to occur in the first place are lacking (Chaudhry, 2017; Denov and Akesson, 2017; Baillie Abidi, 2018; Baillie Abidi and Cleave, 2023). This failure to expose and critique root causes overlooks the significance of the continued impacts of colonialism, particularly for children living in conflict-affected regions (Chaudhry, 2017; Denov and Akesson, 2017; Kindersley and Rolandsen, 2019). Thus, Galtung (1969, 1996) violence triangle offers a helpful framework to understand the multiple layers of violence construction. Engaging young people in this analysis is particularly important given their historical exclusion from participating in conflict analyses and peacebuilding processes.

Equally important to unpacking normalizations of violence with young people is exploring how relations of power influence violence construction and peacebuilding. Lederach (2006) argues that relationships lie at the heart of peacebuilding processes. Given that violence is socially constructed, transforming violence requires a collective and relational approach that recognizes how societies are built on foundations of gender, class, racial, and other forms of inequities. Unfortunately, violence transformation and peacebuilding processes often overlook these foundational inequities, including the exclusion of the voices and meaningful engagement of children and youth, which led Pepper (2017) to argue that age is an additional “vector of oppression” (p. 84). This glaring omission of young people’s voices illustrates how adultism operates in peace and security architectures in a manner that limits our understanding of peace and peace action. Adultism, or the assumption of the inferiority of young people, is supported by a social design that privileges adult perspectives and adults’ power over children and youth (Bell, 1995). This social design skews and limits how we understand children’s lived expertise.

There is increasing recognition of the importance of youths’ participation in peacebuilding, as codified in several UN resolutions, namely Security Council Resolution 2250, which highlights “young people’s inclusive participation and positive contribution to building peace in conflict and post-conflict situations” (United Nations Security Council, 2015, p. 1). However, while youth are forging access to previously adult-controlled political spaces and demonstrating the importance of their participation in peace and security, they continue to largely be excluded from decision-making opportunities (Baillie Abidi, 2018; Berents, 2018; UN, 2022). For example, despite having unique experiences, youth impacted by forced migration describe being “seldom consulted, frequently overlooked, and often unable to fully participate in decision making, the talents, energy, and potential of refugee youth—young people aged 15–24 years old—remain largely untapped” (United Nations High Commissioner for Refugees, 2016, p. 4). Failure to understand youths’ peace and security experiences and perspectives results in a gap in our collective understanding of the complexities, opportunities, and relations of power.

Similarly, children’s experiences are shaped by diverse positionalities and experiences, and yet their lived expertise remains missing from peace and security policy and practice (Baillie Abidi, 2021; Berents, 2015; Bhabba, 2014; Thompson et al., 2019). Despite participation being identified as a right in the Convention on the Rights of the Child (United Nations, 1989), the voices and participation of children are not entrenched in peace and security architectures. The gaps in meaningful engagement of children are often attributed to the lack of appreciation of children as agential members of society (Berents, 2015; Berents and Mollica, 2022; Denov, 2012), stemming from a biased social context that privileges adults (Bell, 1995). Understanding children’s perspectives as incomplete or not valued results in a significant portion of our communities being excluded from peace conceptualizing, planning, and programming, despite recognition that children and youth play an essential role in peacebuilding (McGill and O’Kane, 2015; O’Neil, 2018; Pruitt, 2020; Toh, 2002). Children and youth are central to relational frameworks for peace as they have unique and intersecting needs and perspectives that are often different from adults due to their stage in the life cycle and social conceptualizations of childhood. This research aimed to prioritize children’s perspectives on the multiple dimensions of violence, the important roles of children in violence transformation and peacebuilding, and how centering young people’s visions of peace possibilities within fragile and conflict contexts is an essential part of peace and security.

### Arts-informed research

This qualitative research was inspired by many of the principles of Participatory Action Research (PAR), particularly the importance of centering the lived expertise of people most affected by the social issue being studied, in this case, children and youth affected by armed violence. PAR seeks to engage participants in meaningful ways to inform the research process (Reason and Bradbury, 2008). PAR is action research, designed to create meaningful change *with* participants (MacDonald, 2012), and is an effective methodology to support young people in identifying the changes they want to see in their communities (Baillie Abidi, 2018). This approach recognizes the agency of children and youth, that they are rights holders (UNCRC, 1989), and they are experts of their own diverse lived experiences. This research engaged children and youth to identify how peace and violence exist in their communities, in their relationships, and in their ideas for creating peaceful pathways. Actions based on this research will be further explored in a second phase of the research.

This PAR-inspired research used a range of arts-informed methods to collect and analyze data, while supporting conversations and collective learning, aligning with the critical theoretical and relational framework guiding this research. Utilizing arts-informed methods is an effective approach to engage children and youth in research as it centers relational learning and can destabilize relations of power between researchers and participants (Baillie Abidi, 2018; Shaw et al., 2011). According to Cole and Knowles (2008), arts-informed research “acknowledges the power of art forms to reach diverse audiences and the importance of diverse languages for gaining insights into the complexities of the human experience” (p. 59). Arts-informed methods offer powerful ways for children and youth to demonstrate their perspectives by using visualizations of their experiences and ideas (Guruge et al., 2015), and these methods were particularly effective in this research, given that many young people were learning English. The diverse, non-verbal ways of expressing their perspectives enabled broader and meaningful participation.

The arts-informed methods ranged from using games, poetry, creative writing, and drawing. The focus group agenda flowed from confirming consent and assent, the creation of community guidelines (i.e., rules to guide collective learning to contribute to safer spaces), engaging in community building activities such as exploring what peace means to each participant through a ball toss-peace word association game, reading a poem about children and war called *The Eloquent Young Elephant* (Fitch, 1997), using drawings, creative writing and/or collaging to demonstrate visualizations of their peace learning, and finally, exploring strategies for children and youth to engage in peacebuilding in their homes, schools and communities.

### Methods and participants

Four focus groups were held, two in Cameroon and two in Canada, with children and youth affected by armed violence. The participants ranged in age from 9 to 18, and while all participants fall within the legal definition of a child, some participants preferred to be identified as youth. Thus, throughout the manuscript, the use of children and youth or young people is used to capture this age range.

In Cameroon, the research was conducted in partnership with Local Youth Corner Cameroon, a youth, peace, and security organization leading community-engaged programming with children and youth affected by the two conflicts occurring in the country. Participants were recruited with a poster shared with community partners. One focus group took place in Buea, a region impacted by the Anglophone Crisis, an internal conflict that began in 2017, and the second focus group was held in Yaoundé, the capital city, with children and youth from diverse regions of the country. In total, 31 children and youth participated in the focus groups in November 2024. In Canada, two focus groups were held in December 2024 in collaboration with the YMCA of Greater Halifax/Dartmouth, Immigrant Programs. The participants were recruited with a poster shared through YMCA community programming notifications. A total of 19 newcomer children and youth, representing multiple countries of origin and experiences with varying forms of insecurity and armed violence, participated (Table 1).

**Table 1:** Focus group locations and dates.

Research location	# of participants	Date of workshop
Buea, Cameroon	16	November 2024
Yaoundé, Cameroon	15	November 2024
Halifax, Canada	14	December 2024
Halifax, Canada	5	December 2024

All four focus groups followed a common format starting with introductions and the development of community guidelines, or rules to support building a safer learning experience (e.g., committing to maintaining the confidentiality of stories shared, using respectful language, and endeavoring to be curious instead of judgmental). Following these foundational activities, the participants were invited to pass a ball in a circle while sharing a word they associate with peace as they catch the ball. Words included harmony, no violence, health, safety, freedom, etc. This activity was designed to begin our peace discussions, to get to know each other, and have a little fun, something key to effective engagement with young people (Heartwood Centre for Community Youth Engagement, 2011). For two focus groups where we had more time, one in Cameroon and one in Canada, we also facilitated the “human knot” activity, whereby everyone reaches into the center of a circle and holds hands with two different people. The participants are then asked to unwind without letting go of their partner’s hands. The goal is to form a wider circle holding hands in an uncrossed manner. Following this activity, the children and youth discussed how observation skills, clear communication, listening, testing different strategies, failing and trying again, and teamwork were key to their success, and that these skills are also key to effective peace processes.

The next portion of the focus group involved reading the poem *The Eloquent Young Elephant* written by Fitch (1997), a Canadian poet. The poem focuses on the role of a young elephant who successfully convinces a group of adult elephants not to go to war. After reading the poem together, and reviewing complex words and understanding, which was an important component given the workshop was conducted in English, a learning language for many participants, the participants were invited to reflect on the most meaningful part of the poem and their broader understandings of peace. These discussions led to art-making activities whereby the children and youth could create their own peace representation through poetry, spoken word, drawing, or other methods. Following the artmaking session and discussions about the peace representations, the participants engaged in a discussion about peace actions they see, engage in, or would like to engage in, within their homes, schools, community, and beyond. Establishing ground rules, engaging in ice-breakers, and using creative methods are important research methods that fall in line with the Guidelines for Research with Children and Young People (Shaw et al., 2011).

### Data analysis

The data analysis was derived from all the materials submitted by the participants, including their art representations of peace, which were captured in drawings, comments, and poems; completed handout sheets; and researcher notes recording key comments, insights, and discussions. The researcher engaged in thematic analyses to generate key findings (Braun and Clarke, 2006). Thematic analysis is a helpful approach for research centering on underrepresented voices (Braun and Clarke, 2006, 2024), in this case, that of children and youth affected by armed violence, as it prioritizes participants’ voices in the data. The thematic analysis process involved becoming familiar with the data, including reviewing written and visual data, then generating codes, and finally building themes in alignment with the research questions (Braun and Clarke, 2006, 2024). The reflexive approach also included ongoing debrief discussions among the research team in relation to observations, strategies to mitigate researcher bias, and contextual influences. For example, the Canadian focus groups occurred shortly after the fall of the Bashar al-Assad regime, and given that many of the participants were from Syria, including one of the community partner facilitators, this event informed our discussions on peace, requiring the researcher and community partner to maintain regular check-ins throughout the focus groups.

This research aimed to prioritize the perspectives of children who have experienced armed violence, recognizing the ongoing omission of children’s voices in peace and security dialogs. Due to time and funding limitations, a broader intersectional analysis—exploring how other social identities, such as gender and language, intersect with age and conflict experience—was not included in this phase. The researcher acknowledges that peace and security experiences are shaped by intersecting factors, and the absence of a wider intersectional lens is a limitation, particularly in the context of rising state violence perpetrated against already marginalized communities. Phase two will expand the intersectional analysis with young people affected by armed violence and will be led by youth researchers in collaboration with community partners.

The findings from this phase were largely consistent across the four focus groups. Given the diverse regions represented in Cameroon and the varied countries of origin of participants in Canada, differences between ethnic or national groups were not emphasized to avoid researcher bias or misinterpretation. Moreover, participants were not directly asked to disclose their ethnic group or country of origin, which further limited comparative analysis. The analysis presented here aligns with participatory action research (PAR) principles by centering the voices of children and youth. Phase two will build on this foundation, focusing more explicitly on location as an intersecting factor alongside age, gender, and experiences of armed violence.

### Ethical considerations

This research was guided by an ethics of care, which focused on protecting children’s rights and agency in line with the Guidelines for Research with Children and Young People (Shaw et al., 2011). Ethics approval was awarded through Mount Saint Vincent University (#2024–007).

The ethical guidelines were collaboratively developed with youth working with Local Youth Corner Cameroon and the YMCA. Importantly, the children and youth engaged in this research were already actively involved with the partner organizations, so prior relationships, collaboration, and trust existed.

Parental consent was obtained prior to the initiation of the data collection. Confirmation of assent with the participants was also obtained at multiple points throughout the process, including the beginning, midway, and at the end of the focus group. The community guidelines previously discussed were revisited throughout the focus group to review if everyone was honoring the commitments and if new elements were required to strengthen the safety of the learning experience. Finally, the participants were encouraged to lead the discussion and recommend changes to the agenda, if need be, as the research team was committed to valuing the perspectives of the young people involved.

## Key findings

The aim of this research was to listen and learn with children affected by armed violence about their understanding of peace and children’s roles in creating peace. The following section explores the themes that evolved through discussion and art making with children and youth.

### Children’s peace perspectives

The participants had a lot to say about peace, what it means to them, as well as what it means to be without peace. They described negative and positive conceptualizations of peace and emphasized relations and community as integral elements. For the children and youth involved in this research, peace is about freedom and safety, community, and peace is a process.

#### Peace is freedom and safety

“*Peace is a state in which there is no war and no lack of understanding. Where there is no revenge and no fighting, just calmness*” (research participant). The participants stated that at its core, peace exists when everyone is free from violence and violent structures, when there is “*safety in your country*,” “*no fighting*,” and “*no violence or war*.” They described that peacelessness occurs “*when there is violence, lies, theft, and disobedience*” and this is enabled by “*people that do not want you to have peace*.” In this regard, the children and youth described how corruption, tribalism, leaders who are “*bad people who have no regard for others well-being*,” and the existence of gangs or other armed actors, compromise children’s ability to experience peace, because “*fear dominates*” in these conditions. The children and youth described a range of impacts of collective violence, including loss of life, illness and injury, and a loss of “*calmness*” or mental wellbeing.

When reflecting on the Fitch (1997) poem *The Eloquent Young Elephant*, the participants overwhelmingly chose the stanza that focused on the negative impacts of violence, including the possibility of death as an outcome of war, as the most impactful verse. They reflected on the senselessness of armed violence and the real consequences children are experiencing, questioning the reason why war would be waged at all. During these discussions, participants described facing violence in many contexts, and particularly in Canada, participants described the impact of violent structures, lamenting the need for a “*different approach to policing*,” for example, where young people struggling with the impacts of war were supported instead of punished for trauma-related behaviors. The young participants reflected on how the challenges of armed conflict experiences can influence behavior, leading to cycles of violence, something they wished adult professionals who work with children and youth, namely police officers, health professionals, and teachers, would better understand.

**Figure 1:**
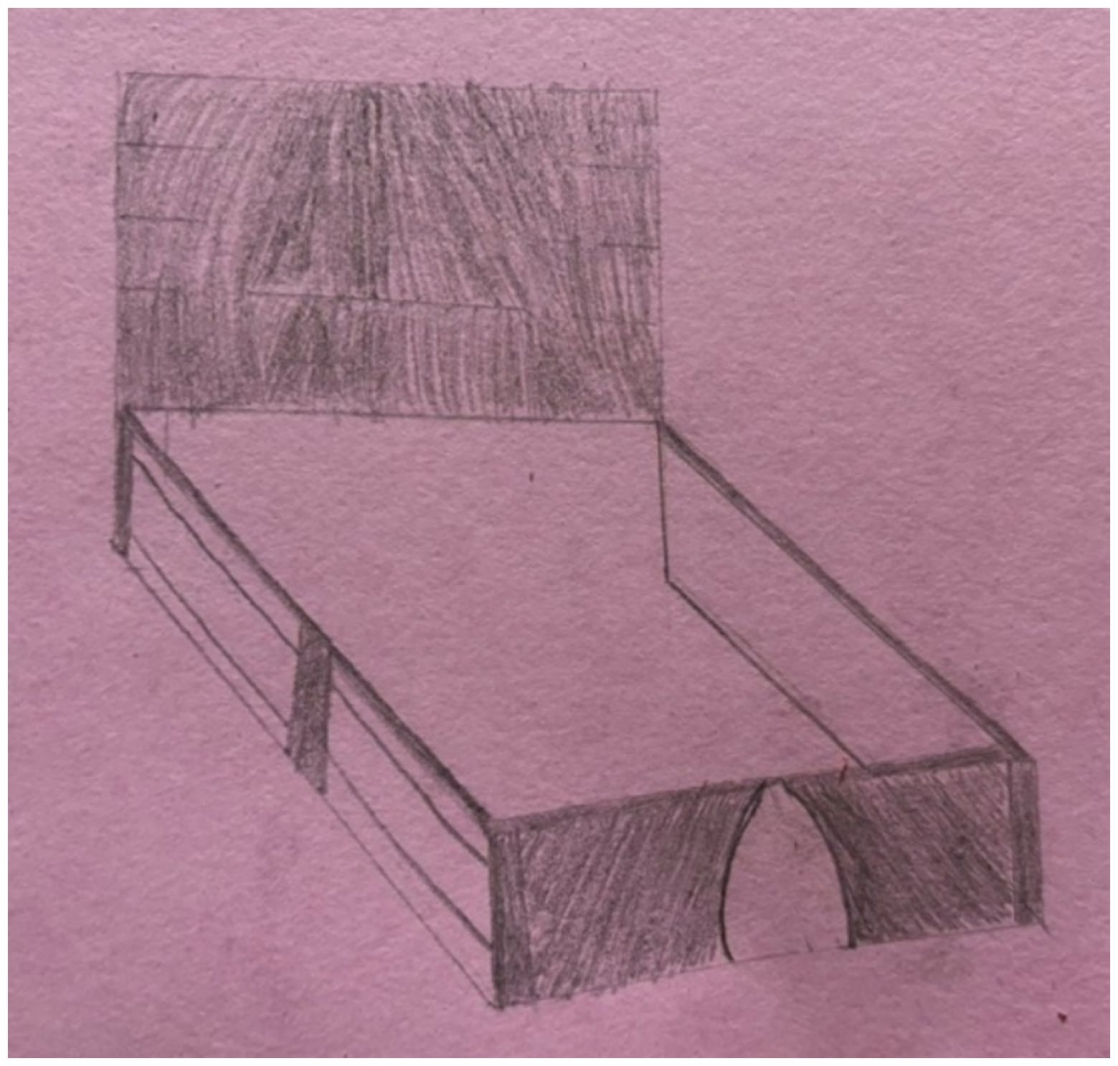
Image of a bed, symbolizing intra, and interpersonal peace; drawn by a Cameroonian participant.

**Figure 2:**
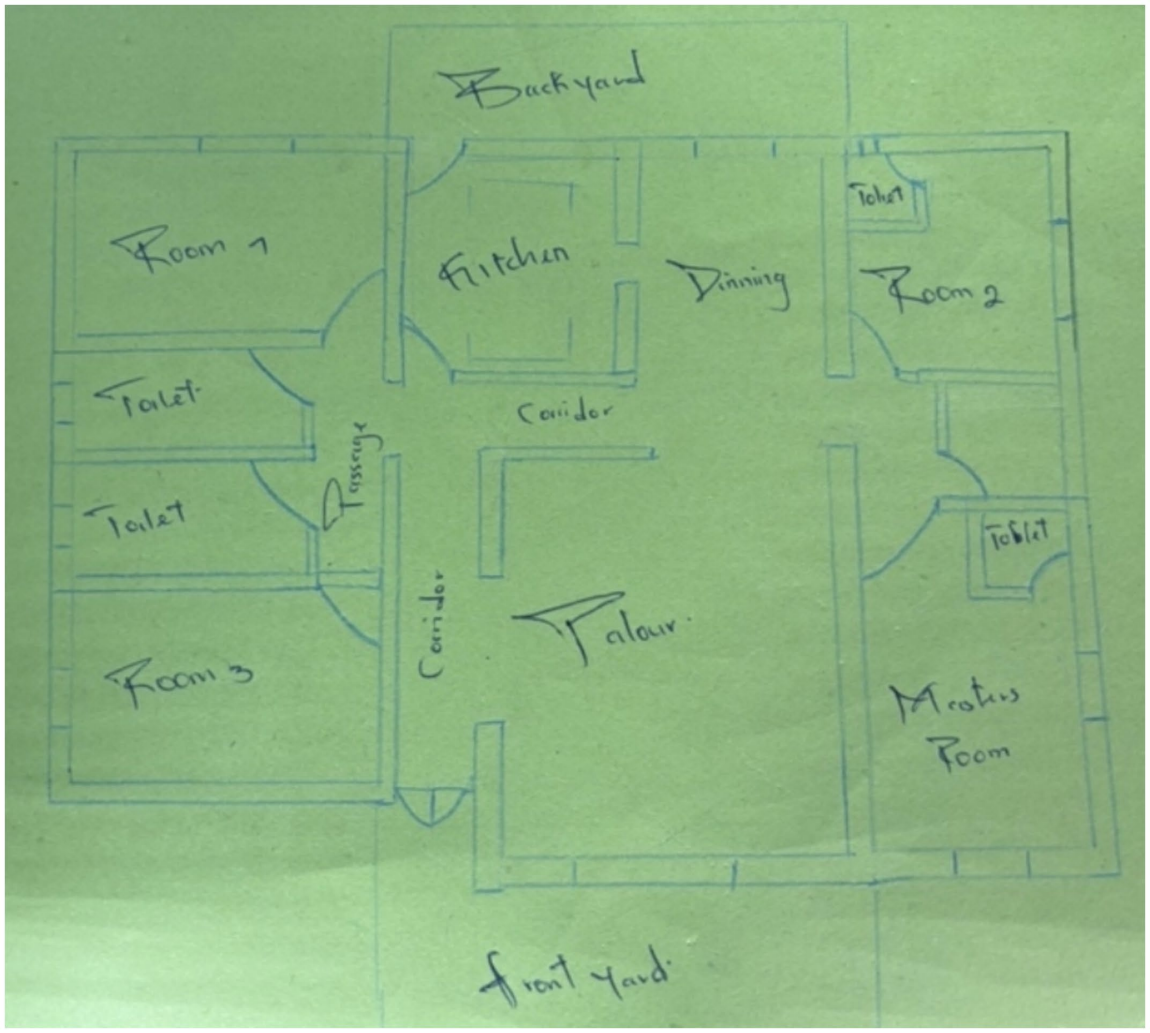
Image of a house blueprint, symbolizing the importance of domestic peace; drawn by a Cameroonian participant.

While all the children and youth involved in this research experienced violence at the socio-political state level, living in a “*safe environment*” for many of the participants equated largely to the interpersonal level, and particularly experiences within the family. The participants discussed family tensions and experiences of “*jealousy*,” “*abuse*,” and neglect as disrupting children’s peace. When invited to create an arts-informed representation of their understanding of peace, several participants focused on interpersonal safety within the family unit and the home. One participant drew a bed and described that after being out in the community where violence is always visible, “*crawling into my bed at night, where I know I am safe, feels peaceful*” (Figure 1). This participant described the importance of their safe family home and how peacefulness for children is generated from the family. Another participant drew a blueprint of a home and described how reducing or preventing community violence will not be possible unless we create peaceful homes where children are protected and cared for (Figure 2). And yet another participant shared:

*In a society where there is a lack of love, there is no peace.*

*In a home full of hate and jealousy there is no peace.*

*In a school full of gossip and jealousy, there is no love or peace.*

*In a neighbor[hood] full of hate and jealousy there is no love or peace.*

*Hence, we should make love the order of the day in order to always ensure peace.*

*Love is peace.*

-Cameroonian participant

These examples demonstrate the essential link between interpersonal and collective violence and the importance of strong and safe family units for children affected by armed violence.

**Figure 3:**
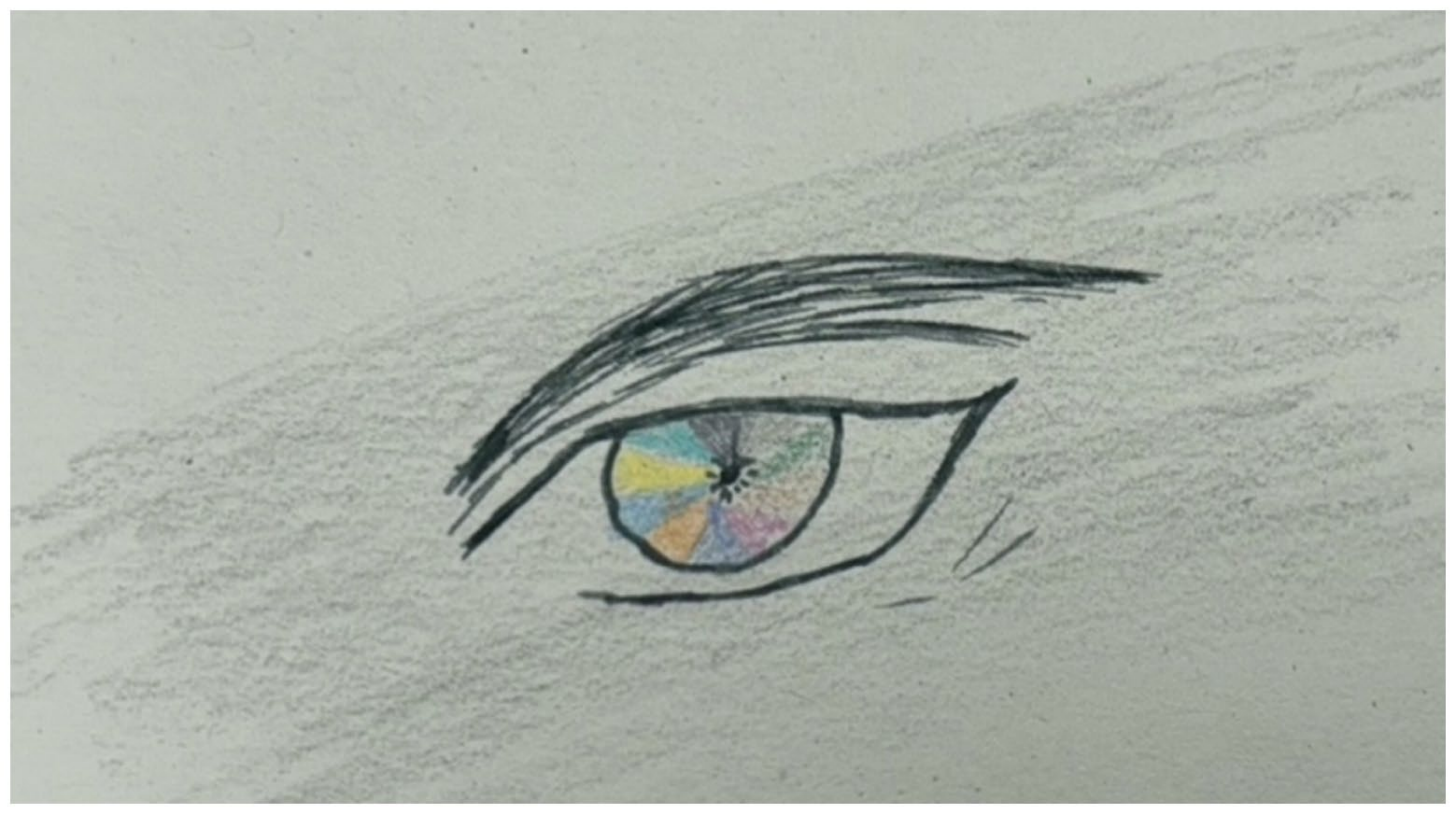
Image of a multi-colored eye, symbolizing respect for diversity; drawn by a Canadian participant.

The participants also highlighted important aspects of positive peace, building equitable and caring social systems, and how freedom and peace are interlinked. One participant argued that peace is present “*when there is freedom and people can do and say what they want*.” The complexities of achieving peace and freedom in a “*violent world*” were thoroughly discussed, with one participant sharing: “*we cannot find peace unless we are free, and we cannot be free until we are at peace.”* Other participants stated that peace occurs when “*everyone is equal and has freedom of speech*,” when we are all living in “*a happy and positive space*,” a “*place where we cannot be judged*,” where we can all “*be yourself*,” and “*when we understand each other*.” For one participant in particular, “*peace is when you love and respect people in your society. Peace makes people have better understanding together*.” In a similar regard, a participant chose to represent their vision of peace, from a positive peace lens, by drawing an eye with a multi-colored iris (Figure 3). They described their image as a representation of valuing diversity and argued that if we celebrate everyone, and appreciate our differences, then peace will be possible.

#### Peace is community

Peace was described as a relational concept, deeply interconnected with community and a sense of belonging. One participant shared how peace is “*a multifaceted concept that encompasses* var*ious aspects of the human experience*” and further argued that the human experience is grounded in relations. Another participant discussed the quintessential role of “*mujtamae”* (community in Arabic), stating, “*when the community is supported, and people can make a better life for their children,*” then peace can be realized. Feeling connected to community, family, and friends was central to the children’s and youth’s peace perspectives. As one participant described, “*there is no peace when you are excluded.”*

Inequities within communities were frequently raised by the participants, who argued that peace can only be achieved when each member of a community is valued. For example, the children and youth in this research suggested that when “*your opinion matters*,” when “*everyone is equal*,” and when there is “*equality between people,”* then peace will be possible. Respecting others, emulating “*trust, honesty and empathy*,” and engaging in “*good communication and collaboration*” create conditions for “*understanding and peace to blossom*.” Community as an important site and source of peace was further demonstrated by a participant who shared an anagram while reflecting on reciprocal peace in relationships:

*PEACE*

*P – practice your moral values instead of just composing them.*

*E – evaluate yourself in a grade of 10 and see how many you score as a peace maker.*

*A – act towards others as you want them to act towards you.*

*C – communicate love, kindness, respect and morality to people around you.*

*E – express yourself with respect towards others because respect is reciprocal.*

- Cameroonian participant

**Figure 4:**
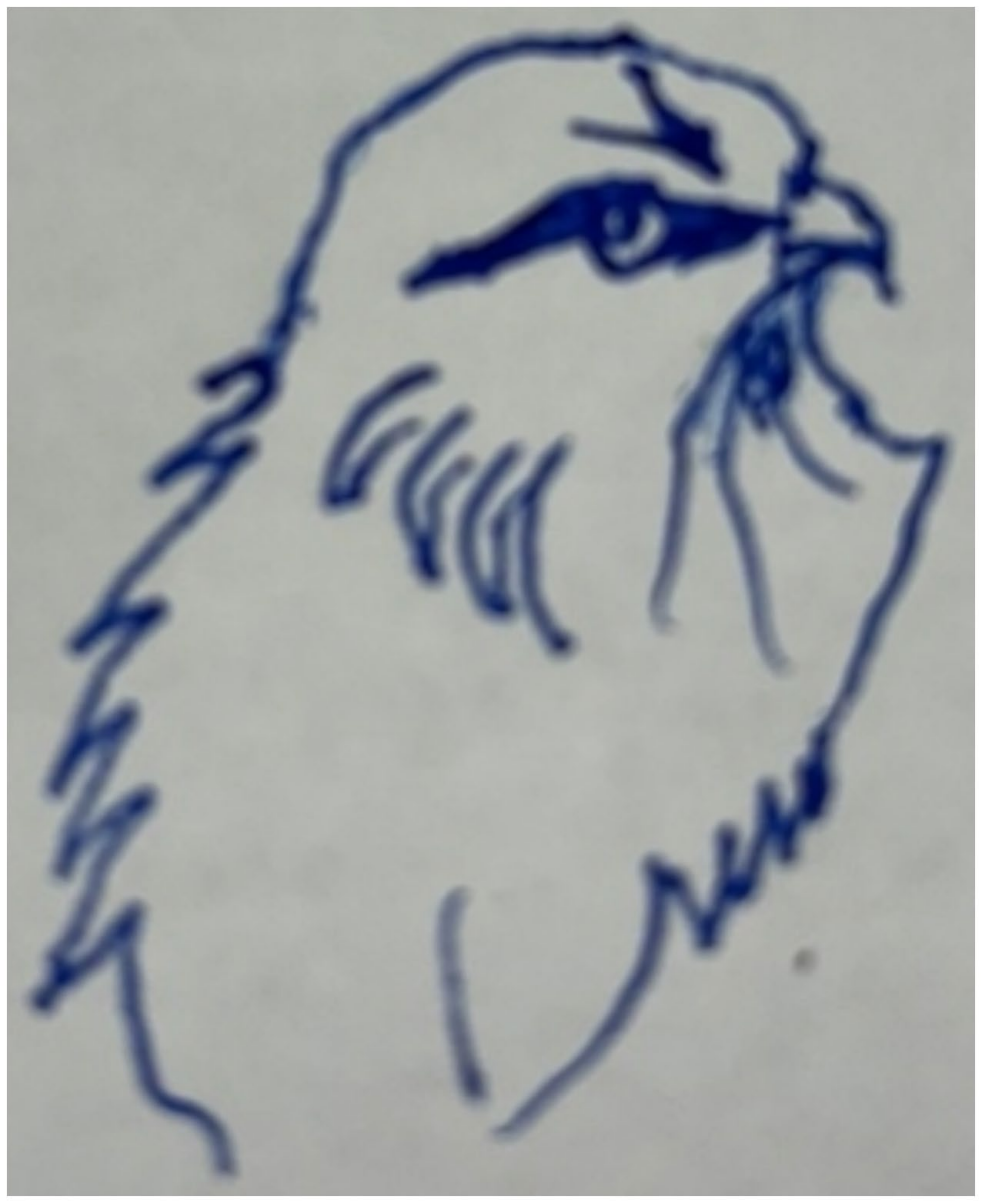
Image of a Serin, a symbol of the Syrian Revolution; drawn by a Canadian participant.

The notion of belonging as being central to peace was demonstrated by another participant’s art representation. The participant drew a Serin, a bird that serves as one of the symbols of the Syrian Revolution (Figure 4). They articulated the impact of violence on their family, from losing loved ones to forced displacement to heightened risks for criminality due to their trauma, and yet the idea of a community of people resisting a violent leader and rallying together to create a peaceful future was comforting and a source of pride for this participant. Several participants, in Cameroon and Canada, discussed how being part of revolutions or communities challenging state-perpetrated violence was an important element of their understanding of peace as they felt part of a larger network of relationships, and they had purpose due to their peace-seeking and collaborative actions.

#### Peace is a process

Beyond describing what peace entails and the notion that peace is built with and in relations, the participants also described peace as a process. One participant shared a Martin Luther King Jr. quote to demonstrate this aspect: “*peace is not merely a distinct goal, but a means by which we arrive at that goal.*” The children and youth in this research described peace as a practice; for some, this practice was connected to faith and prayer, for others it was connected to morals and ethics. One participant articulated, “*peace is achievable, it is not a dream,*” through a poem focused on a conscious journey together:

*Peace Not a Dream*

*Peace not a dream but a reality we weave.*

*A tapestry of love, forgiveness and release.*

*Not just a word but a world to create,*

*Where differences resolve and love participates.*

*Peace not a dream but a choice we make,*

*Every moment, any time for our own sake.*

*Peace not a dream but a journey we take,*

*Together hand in hand for humanity’s sake.*

- Cameroonian participant

This participant further added:

*Be kind to everyone because you never know when you might need them in the future. Be so busy improving yourself that you have no time criticizing others. It’s not very peaceful—like. Remember, we reap what we sow. If you sow peace, you reap peace. Also, put in mind a peaceful environment is a place to be. And remember the way you fix your bed so shall you lie on it. It takes one man to project but a community to spread the finish product so let us come together and spread peace.*

**Figure 5:**
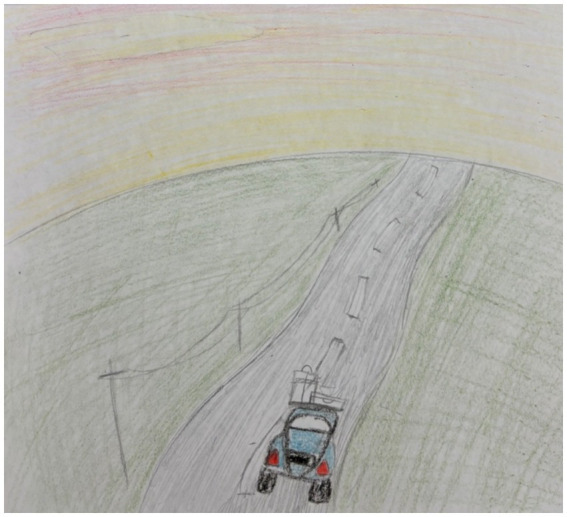
Image of a car on a path, symbolizing a peaceful journey; drawn by a Canadian participant.

Another participant drew a car on a path to demonstrate that each person’s peace journey will be different, and we should each “*choose the path that you want to take in life with no judgement or pressure…it is acceptable to take a different path in life than what you are supposed to do*” (Figure 5).

**Figure 6:**
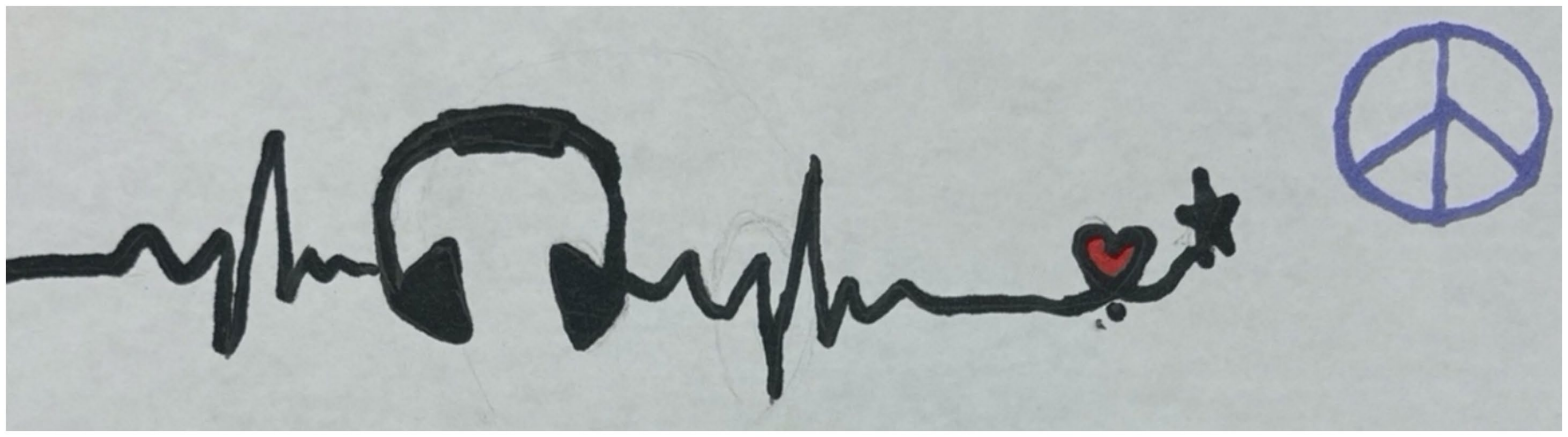
Image of a heartbeat with headphones, symbolizing activities that brings peace; drawn by a Canadian participant.

The participants discussed activities that support peacefulness and “*harmony*” in their lives. Many participants focused on the importance of peace journeys beginning with themselves, building internal “*quiet and serenity*” to flourish in their peace practice. The children and youth in this research shared how music, going to the gym, playing sports, drawing, cooking, playing video games, “*doing something I enjoy*,” and “*taking care of yourself*” are important to begin or sustain a peaceful mental state. For one participant, music was described as central to their feeling of peacefulness, and through music, they can generate peace in their community. They chose to represent their journey of peace by drawing headphones in a heartbeat to symbolize how significant music and art are for their inner peace (Figure 6).

Another participant created a poem to illustrate the importance of inner peace:

*PEACE*

*Peace is a*

*quiet moment.*

*I sit and breathe and let go.*

*My worries fade, my*

*soul glows, the world*

*outside recedes from view*

*and in the silence, I find you.*

*My heart once racing, now at peace,*

*My mind once cluttered now releases.*

*The stillness soothes my soul’s dark night*

*And fills me with a warm, gentle light.*

*In this quiet moment, I am free.*

*My thoughts untangle, clarity I see.*

*No noise, no distractions, just me*

*connected to serenity.*

*So let me sit and breath*

*and be in harmony*

*with peace and glow*

*For in the quiet*

*I find my way.*

- Cameroonian participant

For this participant, inner peace is the starting point to collective peace and as a community, we need to do more to help children find their own sense of peace.

### Children and peacebuilding

While reflecting on *The Eloquent Young Elephant* (Fitch, 1997) and engaging in discussions about what building peace looks like, the children and youth in this research highlighted the important experiences and perspectives that children have in relation to peace and security, especially children affected by armed violence. The participants discussed the essential role of children as peacebuilders in their communities.

#### Wisdom of children

The participants described the importance of respecting and listening to all members of a community, “*no matter your age, your size*” to find a common goal toward peace. One participant shared: “*Peace is all about living together, unity, love and mutual respect. Peace is about giving a listening ear to others [because] everyone’s opinion counts. You are important whether you are big or small, tall or short, fair or dark.”* The children and youth in this research acknowledged their diverse experiences based on their gender, race, proximity to armed violence, age, and education, among other identities and experiences. They also lamented how their experiences are different from those of adults and “*sometimes young people know more than old people.”* The participants in all four focus groups expressed frustration that their lived expertise was not valued, seen, respected, or acted upon; that their wisdom was not recognized in the largely adult-controlled peace and security landscape.

**Figure 7:**
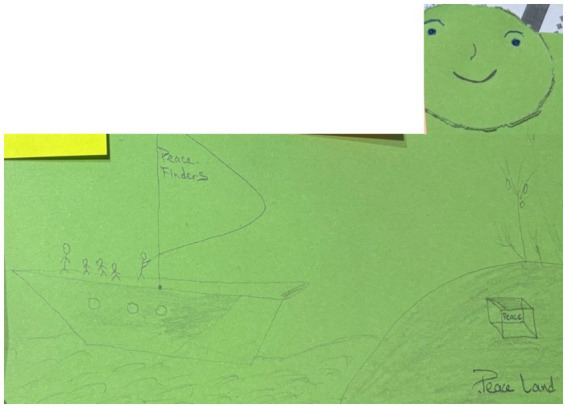
Image of a ship sailing toward “Peace land,” symbolizing children’s peace wisdom; drawn by a participant from Cameroon.

One participant chose to represent children’s knowledge and experience in their peace representation and drew a boat, called the “*Peace Finders*”, an island called “*Peace Land*,” and indicated: “*we are all in this ship on our way to go and mine gold. And our gold here is ‘peace’*” (Figure 7). This participant invited everyone to “*follow us and discover the best way of eradicating war and promoting understanding on our ‘Peace Land’*.” For this young participant, the “us” was children, and the story they shared was one of exceptional navigation skills toward peace because children can see and steer in a different way than adults.

**Figure 8:**
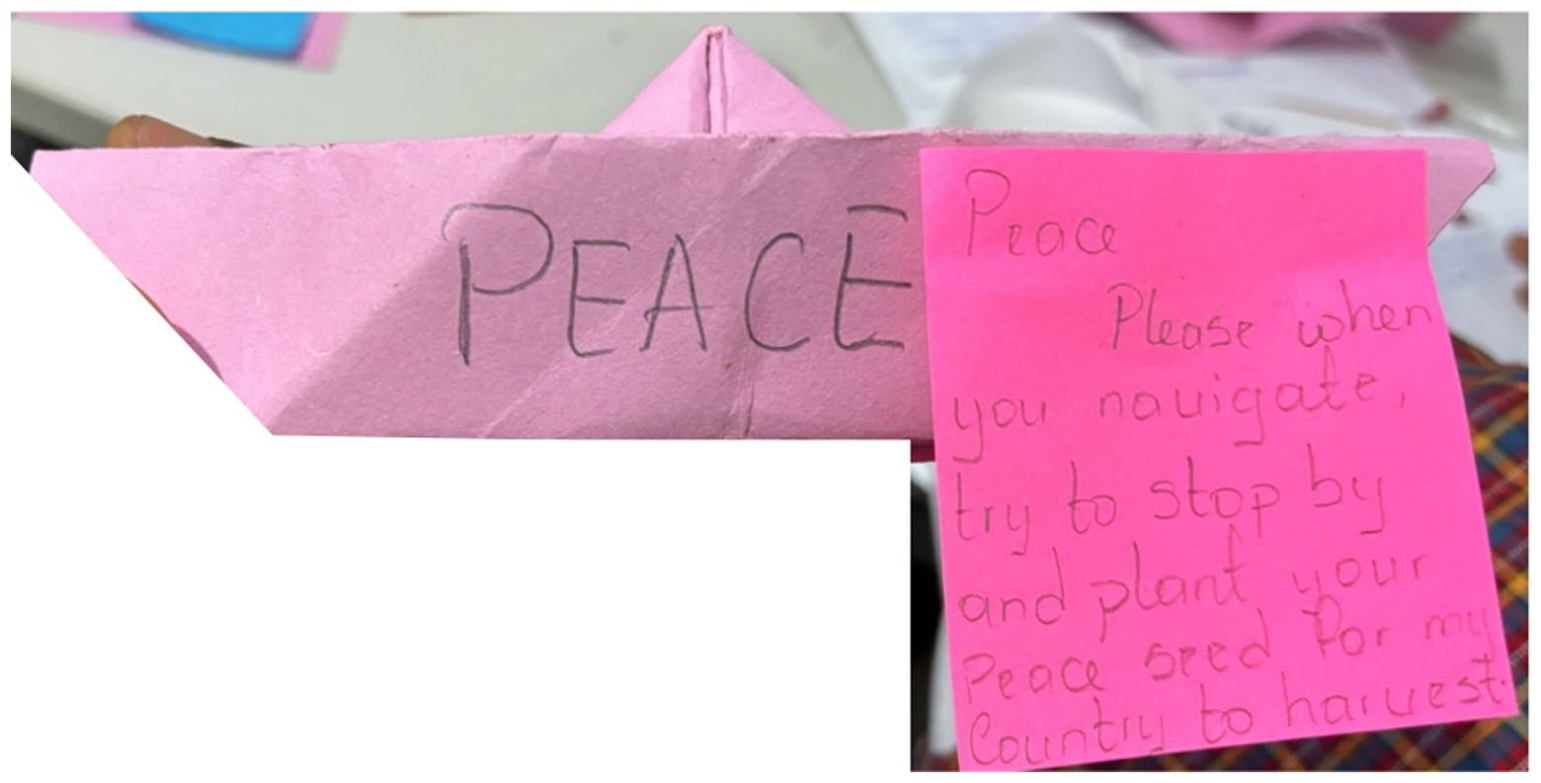
Image of a boat made of paper, symbolizing spreading peace; drawn by a participant from Cameroon.

In reflecting on *The Eloquent Young Elephant* (Fitch, 1997), the participants highlighted the importance of the bravery of the young elephant who shared their perspectives with adults, especially when the adults were preparing to engage in violence. One participant noted: “*the small elephant was brave enough to say his opinion even though they might not listen to him,*” and several participants acknowledged that seeing the bravery of children engaging in peace action—whether in the poem or in real life—can inspire confidence to engage as well. Another participant discussed the importance of sharing knowledge between children and adults, to ensure diverse perspectives are understood, and there is an enhanced opportunity to learn. This participant created a paper boat to symbolize peace, invited adults to “*plant your peace seed for my country to harvest*,” and highlighted how young people can also share their seeds (Figure 8).

#### Children as peacebuilders

Despite their experiences with armed violence, the children and youth in this research demonstrated a strong hope that peace is possible. The participants shared that engaging children who experienced armed violence in peacebuilding is essential to achieving peace and security because they understand the tensions and complexities and have ideas for solutions that may be overlooked by adults or other young people who have never experienced armed violence. A participant stated: “*pain is a universal constant in life. What matters is how or what you do about it. You either crack and break like a pane of glass or rise like a phoenix from its ashes, more powerful than before. Afterall, the purest steel is formed under the greatest pressure*.”

The participants identified several actions that they are already taking or could take in their homes to enhance peace. They discussed supporting and helping with chores, spending quality time with family, and ensuring laughter is part of the process. The participants also discussed the importance of building non-violent and peace communication skills that “*give other people space*” to “*feel heard and accepted*,” use “*our mouths more than our hands to communicate*,” and focus on “*communicating with others to build understanding*.” Overall, the participants discussed how generally “*being respectful*” and remembering to “*love each other*” even during conflict could go a long way to building peace in the home. As one participant argued, *“peace cannot be bought, but it could be practiced.”*

Schools as a site for peacebuilding and peace action were the most commonly discussed area of focus for the participants. The children and youth discussed the importance of having explicit “*peace goals*” at school that prioritized learning peace skills such as peer mediation, non-violent communication, and empathy. The participants discussed that at school, there is a need to strengthen conflict transformation skills to help prevent conflicts among friends from escalating to violence, and shared countless stories of strategies they used to try and resolve conflicts. The strategies ranged from speaking up when witnessing unkind acts or acts of violence, “*saying what you feel to help other people understand you*,” prioritizing inclusion to “*not make some feel excluded*,” modeling respect for different cultures, and working with teachers to strengthen peace in their schools. Some participants were already engaged in school-based peace clubs, and many left the focus groups motivated to start peace clubs in their schools, committing to connect multiple schools in peace actions together. One participant shared that “*building a peace club is one of the goals to achieving peace in our communities, schools, homes, and even our churches and the whole world*” for within these clubs, the central focus is learning how to promote peace, a lifelong and essential skill. The participants expressed confidence in their ability to lead peace in their schools, particularly when they were engaged in group peace actions stemming from peace clubs, and expressed gratitude for the opportunities to lead peace work.

**Figure 9:**
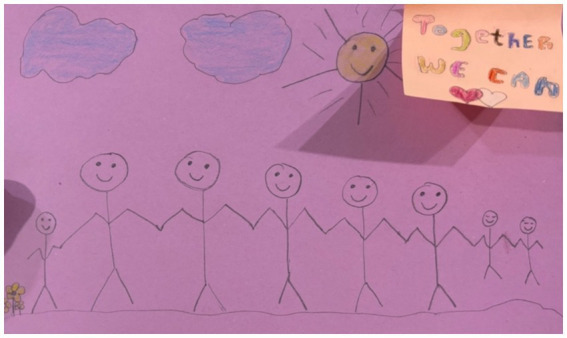
Image of people holding hands, including children; drawn by a Cameroonian participant.

**Figure 10:**
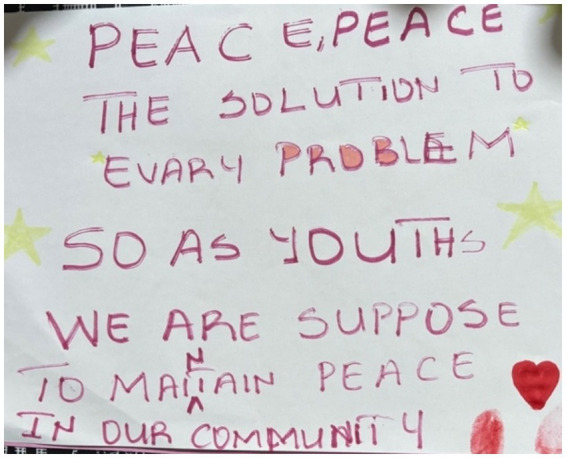
Image of a sign about the role of youth as peacebuilders; drawn by a Cameroonian participant.

Finally, the participants also argued that children are essential to community-based peacebuilding. The children and youth in this research reflected on learning peacebuilding skills from adults in their community and yearned for more intergenerational opportunities to engage in peace work. They expressed a desire to listen and learn with elders and to share insights from younger generations. One participant specifically focused on the intergenerational aspect of peace work by drawing people of all ages holding hands and described the strength of an all-ages—all community member—approach to peace actions (Figure 9). Other participants focused on community action. They spoke of volunteering, helping friends and community members by “*looking out for one another*,” and working on skills to translate experiences from young members of society to elders. Another participant highlighted the capacity of young people to lead peace, arguing that young people have responsibilities to create and sustain peace in communities (Figure 10). The participants expressed that peace cooperatives, focused on building community peace skills, would be an important next step.

Children and youth have a lot to share about peace and their role in peacebuilding. The key findings from this research demonstrate the importance of valuing the lived expertise of children and youth affected by armed violence to build more comprehensive and effective peace and security frameworks.

## Discussion and recommendations

The opening overview of the UN annual report on children and armed conflict states: “violence against children in armed conflict reached unprecedented levels… Children bore the brunt of relentless hostilities and indiscriminate attacks, and were affected by the disregard for ceasefires and peace agreements and by deepening humanitarian crises” (United Nations Secretary-General, 2025, p. 2). From Gaza to the Democratic Republic of Congo to Haiti, children are suffering extreme consequences in fragile and conflict-affected contexts, something Machel (1996) has been asserting for over 30 years. Reflecting on the possibilities for violence transformation, Machel (1996) stated:

Children can help. In a world of diversity and disparity, children are a unifying force capable of bringing people to common ethical grounds. Children’s needs and aspirations cut across all ideologies and cultures. The needs of all children are the same: nutritious food, adequate health care, a decent education, shelter, and a secure and loving family. Children are both our reason to struggle to eliminate the worst aspects of warfare and our best hope for succeeding at it.

Given the state of the world, one has to wonder if the global community really believes that children are a “unifying force.” Despite being increasingly and disproportionately impacted by armed violence (United Nations Secretary-General, 2025; World Health Organization, 2014), children continue to be framed as a peripheral issue in peace and security. In fact, many peace agreements fail to mention children at all (Grace, 2024).

As armed conflicts grow in number and intensity globally, the need to understand the root causes of armed violence, particularly the structural and cultural forms of violence that enable direct violence to occur (Galtung, 1996), becomes paramount. Watchlist on Children and Armed Conflict is calling for “UN agencies, civil society organizations, humanitarian organizations, and relevant academic institutions” to invest in better understanding the root causes of armed conflict as a strategy “to inform future responses to grave violations against children” (Grace, 2024). To do this conflict analysis work effectively, and violence prevention work particularly, we need to understand the experiences of those most impacted, children and youth. The young people engaged in this research clearly articulated why children’s and youth’s perspectives on violence, peace, and security are essential. First, they identified the importance of the interconnections between intrapersonal, interpersonal, and collective violence, and how a “*safe environment*” in the home, family, school, and community is essential to sustainable peace. Research continues to underscore the broad risks of interpersonal violence, from impacts on individual health and wellbeing to normalizing violence within communities (Mercy et al., 2017; World Health Organization, 2014). Moreover, in global peace and security architectures, which are informed almost exclusively by adult men, priority is largely attributed to collective violence, often minimizing the impact and influence of interpersonal violence. Failing to see the strings that bind interpersonal and collective violence has contributed to maintaining systems of cultural violence that are gendered and not child-responsive.

Second, the children and youth articulate, through their lived expertise as young people affected by armed violence, how violence prevention and peacebuilding are predicated on building equitable communities. They describe the need for culturally responsive communities, where *“everyone is equal,”* seen, heard, and valued, and how these are the conditions for “*understanding and peace to blossom*.” The children’s and youths’ perspectives align with Lederach (2006) emphasis on relationships as being central to peacebuilding. They also align with the Sustainable Development Goals (UN, 2015), which emphasize equity and social justice as being quintessential to peace and development. All 17 goals connect with the idea of equitable communities; however, goal 16, Peace, Justice, and Strong Institutions, particularly recognizes how violence ruptures peace possibilities and that the “protection of fundamental freedoms” and the enforcement of “non-discriminatory laws and policies” are important cornerstones for peace (ibid). For the young people involved in this research, the “we” is essential, and peace through community is demonstrated in enhanced empathy for each other, including children who may exhibit trauma-related behaviors that violate community norms. For example, building justice, educational, and social policy and practices that recognize the complex experiences of children affected by armed conflict was identified by the children and youth as an important approach to strengthening communities. The ideal community illustrated by the young people in this research would work together toward peaceful solutions where our collective humanity is of utmost importance.

Finally, the children and youth in this research described peace as a process that they are actively influencing in many ways, but are often denied access to at the formal and State levels. Children’s place in peace and security has historically been tied to the notions of victimhood and vulnerability (Berents, 2015; Berents and Mollica, 2022; Denov, 2012), demonstrated in mechanisms that are designed to protect children from adult-perpetrated violence or in the politicization of childhood. While images of and narratives about children are regularly used to generate political support for war and security actions (Berents, 2020; Beier, 2015), rarely are children the true source of the conflict, and even more rarely they are included in peace processes. Adultism, which orients age as a “vector of oppression” (Pepper, 2017), minimizes a holistic understanding of peace and security, and contributes to actions that lack the perspectives of the most disproportionately impacted members of our communities, children and youth. Despite these barriers, children are already building peace and expressing a desire to learn, develop, and enhance peace practices, such as non-violent communication. As one participant shared, “*peace not a dream but a reality we weave*,” the children and youth expressed a desire to actively engage in peacebuilding through learning from elders, and from each other. Failing to engage children in peacebuilding leads to the exclusion of their important perspectives, misses opportunities for intergenerational learning, and reduces peace-related skills development.

Thus, based on the insights and perspectives of 50 children and youth engaged in this research, the following recommendations are put forth for further consideration:

### Value children’s and youths’ peace perspectives

- Challenge adult-centric ways of knowing and doing by recognizing children and youth as equals in peacebuilding.
- Listen and learn from and *with* children and youth affected by armed violence, in different regions and with diverse social positionalities and lived expertise, to fill gaps in understanding of violence normalization processes, young people’s needs and their visions for peace strategies.
- Explore the connections between and impacts of interpersonal and collective violence *on* children and youth.
- Develop professional development opportunities for adults who work with children and youth—in consultation *with* children and youth affected by armed violence—for example, police, teachers, and health professionals, to enhance understanding of young people’s needs and agency.
- Build research skills *with* young people so they are informing and influencing peace and security research questions, methodologies, analysis, and recommendations for future research, policy, and practice.

### Building peace with children and youth

- Strengthen community social safety nets and provide support for children and youth affected by armed violence based on their identified needs.
- Create intergenerational learning opportunities to teach critical thinking and peacebuilding skills to and *with* children and youth.
- Empower children and youth, particularly those affected by armed violence, to connect with other young people to engage meaningfully in peace action in their schools and communities by providing adult partnerships and material resources.
- Empower children and youth, particularly those affected by armed violence, to meaningfully engage in developing child-responsive peace and security policy and practice that challenges normative frameworks of normalized violence and strengthens the impact of the peacebuilding work.
- Enhance respect for children’s rights and existing protection mechanisms through community education and political accountability mechanisms.
- Advocate for a Children, Peace, and Security agenda to demonstrate the centrality of children to long-term peace and security and build mechanisms to advance peace collaboratively.

## Conclusion

The state of the world today is fraught with images of violence and intensifying inequity. We are living in a time of existential threats and harms that are disproportionately impacting young people. Now more than ever is the time to learn from and *with* children and youth to understand how they see the world around them and peace possibilities. In total, 50 young people affected by armed violence participated in this research, sharing their ideas of what peace entails, how we can build and sustain peace, and why children and youth are key to strengthening the peace and security architecture. Conceptualizations of effective peace and security must prioritize meaningful engagement with young people through listening, learning, and collaboration, recognizing that children are not incomplete or merely in the process of becoming agents, but are already significant actors within their communities.

As we celebrate the tenth anniversary of the Youth, Peace and Security Agenda, an agenda built on the understanding of the importance of youths’ participation in peace and security, it is important to reflect on the absence of an equivalent agenda for children. A Children, Peace and Security (CPS) Agenda that recognizes the unique needs, experiences, and perspectives of children is needed to advance protections for and participation in peace and security policy and practice. Building a CPS Agenda requires the intentional and thoughtful engagement of children. This research sought to ensure the diverse lived expertise of fifty children and youth affected by armed violence is seen, heard, and valued, and that their peace insights, as those most impacted by peacelessness, are amplified and centered. Creating sustainable peace is a complex journey, one that requires respect for shared humanity and collaboration across differences. As this wise Canadian participant shared, “*peace is what we all need*.”

*Peace is what we all need.*

*A calm place for hearts to feed.*

*No more fighting, no more pain.*

*Only sunshine after rain.*

*Let us work to understand,*

*Reach out with helping hand.*

*In our hearts, lets plant seeds,*

*Of peace, of love, of what we need.*

*Together strong, we can stand*

*Creating peace across the land.*

## Data Availability

The datasets presented in this article are not readily available because personal information is protected by limiting its use and disclosure to what is necessary and consented to. Requests to access the datasets should be directed to Catherine Baillie Abidi, catherine.baillieabidi@msvu.ca.
