# Supplementary material for: “There is no peace when you are excluded”: exploring peace and peacebuilding with children and youth affected by armed violence
Source: Front Psychol. 2025 Nov 18;16:1689758. doi: 10.3389/fpsyg.2025.1689758 (PMC12670274; doi:10.3389/fpsyg.2025.1689758)
Supplement: Supplementary file 1 [file Image_1.pdf]

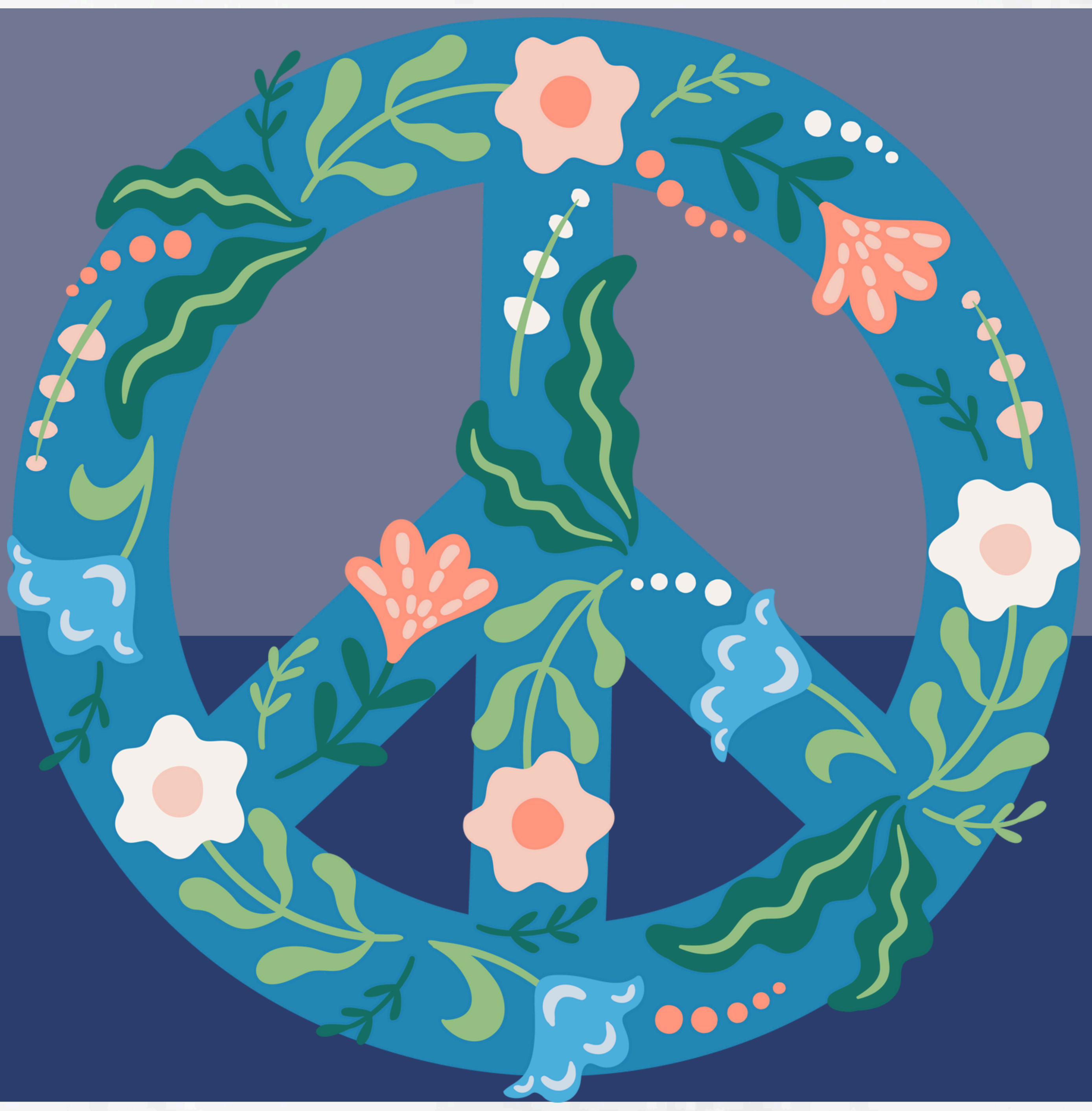

Seeking youth participants for a research study

Researchers from Mount Saint Vincent University (Canada), YMCA Immigrant Centre (Canada), and Local Youth Corner (Cameroon) are conducting a study to know more about **how children and youths define peace**, **how they promote peace in their communities, and gather their creative recommendations to achieve peace.**

If you are...

- between 9-18 years old
- living in Canada or Cameroon
- involved in programming with Local Youth Corner Cameroon or YMCA Immigrant Programs

then you can participate in the study. This is an opportunity for your voice to be heard as we come together and discuss your thoughts about peace, the importance of your role in peacebullding and share ideas on how we can achieve peace in your own communities.

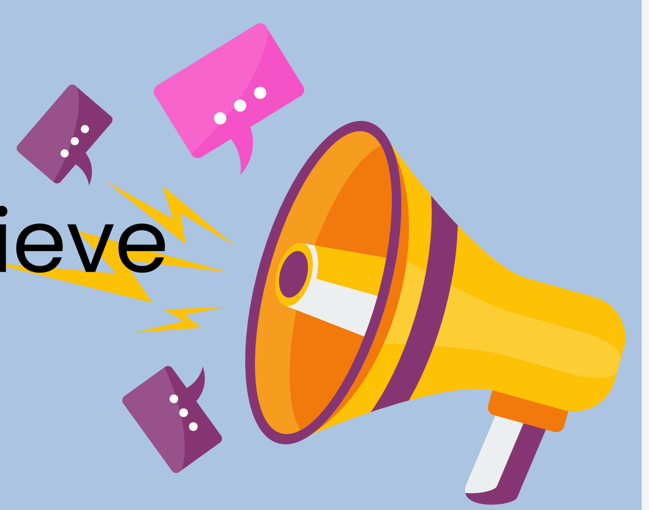

What will we be doing?

Participants will be asked to join 2-3 in-person focus groups where you will be invited to reflect on what peace means and your role as peace builders. You will be invited to share your reflections through story-telling, drama and poetry. These focus groups will result in the development of a Peace Curriculum celebrating the ideas and experiences of children and youth.

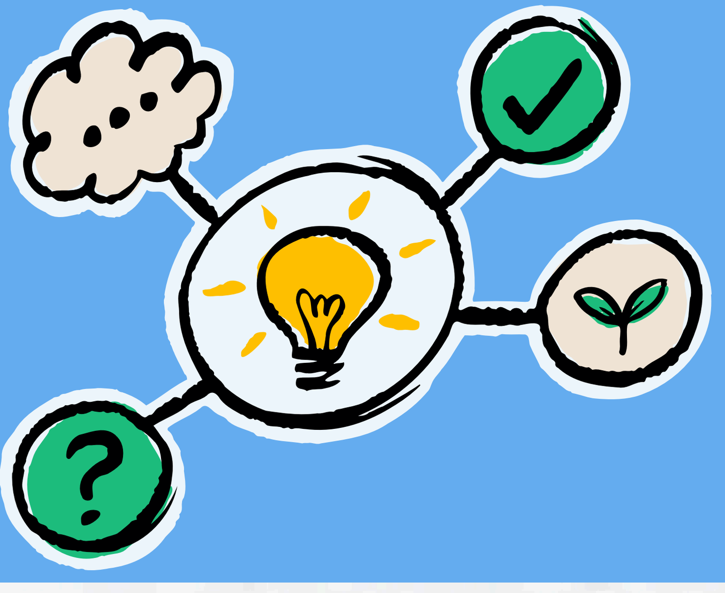

If you are interested in participating or would like to know more information, please email:

For Canada: Dr Catherine Baillie Abidi (Catherine.baillieabidi@msvu.ca) and Tara Liscano (tara.liscano@msvu.ca)
For Cameroon: Mansuru Usmanu Mohmukwe (Us.mansuru@loyocameroon.org)

MOUNT SAINT VINCENT UNIVERSITY
